# 1-*tert*-Butyl 2-ethyl 5-chloro-3-(2-furo­yl)-1*H*-indole-1,2-dicarboxyl­ate

**DOI:** 10.1107/S1600536813005059

**Published:** 2013-02-28

**Authors:** Mohammad Hassam, Vincent J. Smith

**Affiliations:** a Department of Chemistry and Polymer Science, University of Stellenbosch, Private Bag X1, Matieland 7602, South Africa

## Abstract

In the title compound, C_21_H_20_ClNO_6_, the furan moiety is located above the mean plane of the indole ring and displays rotational disorder (*i.e.* rotation through 180°); the site occupancy of the major component is 0.809 (6). In the crystal, C—H⋯O inter­actions link the mol­ecules into chains which run parallel to the *b* axis.

## Related literature

For background to the use of indoles as scaffolds in the synthesis of HIV-agents, see: Hassam *et al.* (2012 ▶) and for a recent review on stages of non-nucleoside reverse transcriptase inhibitors, see: Reynolds *et al.* (2012 ▶). For the crystal structures of closely related compounds, see: Beddoes *et al.* (1986 ▶), Hassam & Smith (2012 ▶, 2013 ▶).
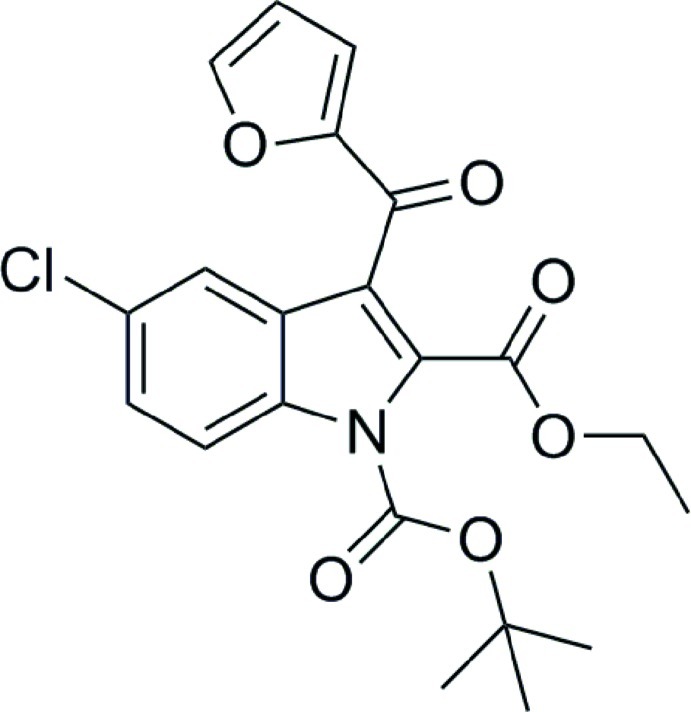


## Experimental

### 

#### Crystal data

C_21_H_20_ClNO_6_
*M* *_r_* = 417.83
Monoclinic, 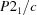

*a* = 9.8354 (8) Å
*b* = 8.0938 (7) Å
*c* = 25.435 (2) Å
β = 99.344 (1)°
*V* = 1997.9 (3) Å^3^
*Z* = 4
Mo *K*α radiation
μ = 0.23 mm^−1^
*T* = 100 K
0.32 × 0.32 × 0.24 mm

#### Data collection

Bruker APEXII CCD diffractometer
Absorption correction: multi-scan (*SADABS*; Bruker, 2009 ▶) *T*_min_ = 0.930, *T*_max_ = 0.947
11690 measured reflections
4483 independent reflections
3877 reflections with *I* > 2σ(*I*)
*R*_int_ = 0.019

#### Refinement

*R*[*F* ^2^ > 2σ(*F* ^2^)] = 0.034
*wR*(*F* ^2^) = 0.084
*S* = 1.05
4483 reflections
279 parameters
8 restraints
H-atom parameters constrained
Δρ_max_ = 0.29 e Å^−3^
Δρ_min_ = −0.38 e Å^−3^

### 

Data collection: *APEX2* (Bruker, 2009 ▶); cell refinement: *SAINT* (Bruker, 2009 ▶); data reduction: *SAINT*; program(s) used to solve structure: *SHELXS97* (Sheldrick, 2008 ▶); program(s) used to refine structure: *SHELXL97* (Sheldrick, 2008 ▶); molecular graphics: *X-SEED* (Barbour, 2001 ▶; Atwood & Barbour, 2003 ▶); software used to prepare material for publication: *X-SEED*.

## Supplementary Material

Crystal structure: contains datablock(s) I, global. DOI: 10.1107/S1600536813005059/zl2535sup1.cif

Structure factors: contains datablock(s) I. DOI: 10.1107/S1600536813005059/zl2535Isup2.hkl

Supplementary material file. DOI: 10.1107/S1600536813005059/zl2535Isup3.cml

Additional supplementary materials:  crystallographic information; 3D view; checkCIF report

## Figures and Tables

**Table 1:** Hydrogen-bond geometry (Å, °)

*D*—H⋯*A*	*D*—H	H⋯*A*	*D*⋯*A*	*D*—H⋯*A*
C13*A*—H13*A*⋯O5^i^	0.95	2.37	3.187 (3)	144

Symmetry code: (i) 
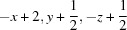
.

**Fig. 1.**
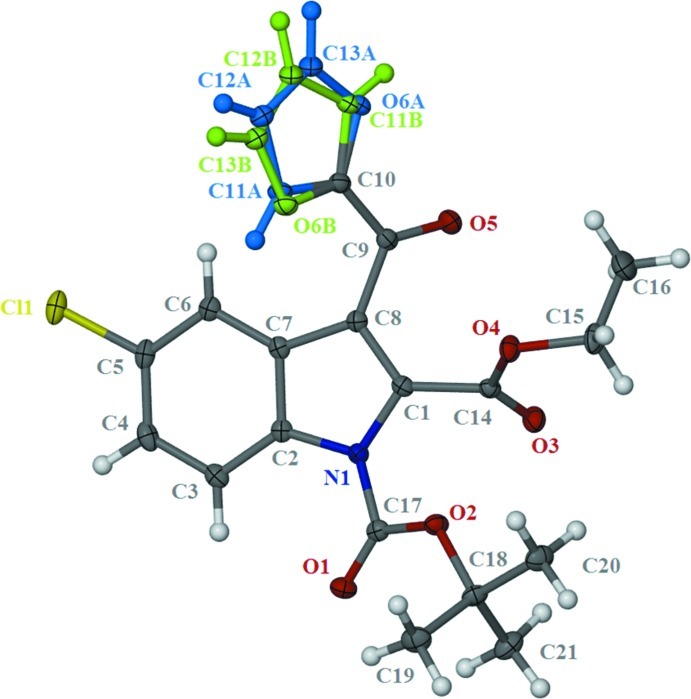
The molecular structure of the title compound showing the atom numbering scheme and the displacement ellipsoids drawn at the 50% probability level. The disorder of the furan moiety is shown different colours, major (blue) and minor (green).

**Fig. 2.**
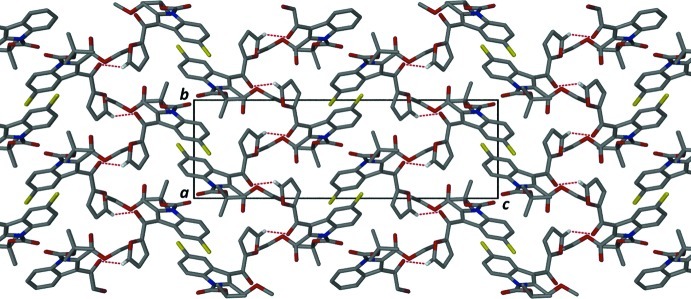
The diagram shows the C—H···O hydrogen bond chains which propagate parallel to the b axis.

## supplementary crystallographic information

### Comment

Ethyl-5-chloro-1*H*-indole-2-carboxylate has been employed as a building block in the synthesis of various anti-HIV active molecules particularly in the search for novel non-nucleoside reverse transcriptase inhibitors (Hassam *et al.* 2012, Reynolds *et al.* 2012). Protection on the indole NH of ethyl 5-chloro-3-(2-furoyl)-1*H*-indole-2-carboxylate was carried out with di-*tert*-butyl-dicarbonate using 4-dimethylaminopyridine as a catalytic base.

The title compound, C_21_H_20_ClNO_6_, crystallizes with one molecule in the asymmetric unit (Fig. 1). The furan group is disordered over two positions with major (A) and minor (B) components in a 0.809 (6):0.191 (6) ratio. The dihedral angles between the mean planes of the 5-chloro indole ring (Cl1/N1/C1—C8) and the disordered furan rings (O6A/C10/C11A/C13A and O6B/C10/C11B/C13B) are 58.01 (13)° and 59.70 (68)°, respectively. The angles between the mean planes of the indole ring and the *N*-*tert*-butyloxy, ethyl ester and the ketone groups are 26.90 (04)°, 55.71 (04)° and 46.18 (05)°, respectively. The torsion angles of O5/C9/C10/O6A and O5/C9/C10/O6B are 12.1 (3)° and -157.5 (1)°, respectively, thereby describing the major component in a *cis* conformation and the minor component in a *trans* conformation. Molecular packing shows the molecules being connected *via* weak C13A—H13A···O5 intermolecular interactions that link molecules into chains which run parallel to the *b* axis (Fig. 2, Table 1).

### Experimental

A catalytic amount of 4-dimethylaminopyridine was added to a solution of ethyl 5-chloro-3-(2-furoyl)-1*H*-indole-2-carboxylate (1.00 g, 3.14 mmol) in THF (20 ml), followed by the addition of di-*tert*-butyl dicarbonate (1.10 g, 5.04 mmol). The reaction mixture was then stirred at room temperature for 2 h. The solvent was removed under vacuum and the resulting residue was recrystallized from a hexane/dichloromethane solvent (4:1) to obtain the title compound as a colourless crystalline material (1.14 g, 87%). ^1^H NMR (300 MHz, CDCl_3_) *δ* 8.08 (dd, *J* = 9.0, 0.5 Hz, 1H), 7.74 (dd, *J* = 2.1, 0.5 Hz, 1H), 7.65 (dd, *J* = 1.7, 0.8 Hz, 1H), 7.39 (dd, *J* = 9.0, 2.1 H, 1H), 7.20 (dd, *J* = 3.6, 0.8 Hz, 1H), 6.59 (dd, *J* = 3.6, 1.7 H, 1H), 4.17 (q, *J* = 7.2 Hz, 2H), 1.63 (s, 9H), 1.15 (t, *J* = 7.2 Hz, 3H). ^13^C NMR (75 Hz, CDCl_3_) 177.17, 161.30, 152.79, 148. 39, 147.33, 134.24, 133.16, 130.17, 127.59, 127.36, 121.28, 120.65, 119.81, 116.35, 112.80, 86.62, 62.43, 27.87, 13.78. HRMS calculated for C_21_H_21_ClNO_6_ [*M*+H]^+^, 418.1057 found 418.53.

### Refinement

All non-hydrogen atoms were refined anisotropically. H atoms were placed geometrically [C—H = 0.95 - 0.99 Å; with *U*_iso_(H) = 1.2 - 1.5Ueq(C)] and constrained to ride on their parent atoms. The site-occupancy factors of the disordered thiophene moieties were initially set to 0.5 and then refined, leading to an occupancy of 0.809 (6) and 0.191 (6) for the major and minor components, respectively. Bond lengths for the furan and phenyl moieties were restrained to be similar (s.u. = 0.002 Å). Atom displacement parameters for overlaping atoms of the disordered models were constrained to be each identical.

### Crystal data

**Table:**

C_21_H_20_ClNO_6_	*F*(000) = 872
*M**_r_* = 417.83	*D*_x_ = 1.389 Mg m^−^^3^
Monoclinic, *P*2_1_/*c*	Melting point: 378(2) K
Hall symbol: -P 2ybc	Mo *K*α radiation, λ = 0.71073 Å
*a* = 9.8354 (8) Å	Cell parameters from 5304 reflections
*b* = 8.0938 (7) Å	θ = 2.4–27.9°
*c* = 25.435 (2) Å	µ = 0.23 mm^−^^1^
β = 99.344 (1)°	*T* = 100 K
*V* = 1997.9 (3) Å^3^	Square block, colourless
*Z* = 4	0.32 × 0.32 × 0.24 mm

### Data collection

**Table:**

Bruker APEXII CCD diffractometer	4483 independent reflections
Radiation source: fine-focus sealed tube, Bruker SMART Apex	3877 reflections with *I* > 2σ(*I*)
Graphite monochromator	*R*_int_ = 0.019
φ and ω scans	θ_max_ = 28.2°, θ_min_ = 1.6°
Absorption correction: multi-scan (*SADABS*; Bruker, 2009)	*h* = −12→13
*T*_min_ = 0.930, *T*_max_ = 0.947	*k* = −10→4
11690 measured reflections	*l* = −31→33

### Refinement

**Table:**

Refinement on *F*^2^	Primary atom site location: structure-invariant direct methods
Least-squares matrix: full	Secondary atom site location: difference Fourier map
*R*[*F*^2^ > 2σ(*F*^2^)] = 0.034	Hydrogen site location: inferred from neighbouring sites
*wR*(*F*^2^) = 0.084	H-atom parameters constrained
*S* = 1.05	*w* = 1/[σ^2^(*F*_o_^2^) + (0.0359*P*)^2^ + 0.8831*P*] where *P* = (*F*_o_^2^ + 2*F*_c_^2^)/3
4483 reflections	(Δ/σ)_max_ = 0.001
279 parameters	Δρ_max_ = 0.29 e Å^−^^3^
8 restraints	Δρ_min_ = −0.38 e Å^−^^3^

### Special details

**Table:**

Geometry. All e.s.d.'s (except the e.s.d. in the dihedral angle between two l.s. planes) are estimated using the full covariance matrix. The cell e.s.d.'s are taken into account individually in the estimation of e.s.d.'s in distances, angles and torsion angles; correlations between e.s.d.'s in cell parameters are only used when they are defined by crystal symmetry. An approximate (isotropic) treatment of cell e.s.d.'s is used for estimating e.s.d.'s involving l.s. planes.
Refinement. Refinement of *F*^2^ against ALL reflections. The weighted *R*-factor *wR* and goodness of fit *S* are based on *F*^2^, conventional *R*-factors *R* are based on *F*, with *F* set to zero for negative *F*^2^. The threshold expression of *F*^2^ > σ(*F*^2^) is used only for calculating *R*-factors(gt) *etc*. and is not relevant to the choice of reflections for refinement. *R*-factors based on *F*^2^ are statistically about twice as large as those based on *F*, and *R*- factors based on ALL data will be even larger.

### Fractional atomic coordinates and isotropic or equivalent isotropic displacement parameters (Å^2^)

**Table:**

	*x*	*y*	*z*	*U*_iso_*/*U*_eq_	Occ. (<1)
Cl1	0.77842 (4)	0.54454 (4)	−0.051345 (13)	0.02592 (10)	
O1	0.25661 (9)	0.04213 (12)	0.01491 (4)	0.0207 (2)	
N1	0.45710 (11)	0.11938 (13)	0.06875 (4)	0.0152 (2)	
C1	0.53993 (13)	0.12814 (16)	0.11858 (5)	0.0157 (3)	
O2	0.27094 (9)	0.06519 (12)	0.10504 (3)	0.0189 (2)	
C2	0.51962 (13)	0.21382 (16)	0.03288 (5)	0.0157 (2)	
O3	0.50752 (10)	−0.12594 (12)	0.16190 (4)	0.0223 (2)	
C3	0.47829 (14)	0.24137 (16)	−0.02143 (5)	0.0190 (3)	
H3	0.3964	0.1936	−0.0403	0.023*	
O4	0.51187 (10)	0.11461 (12)	0.20738 (3)	0.0206 (2)	
C4	0.56192 (13)	0.34160 (16)	−0.04673 (5)	0.0214 (3)	
H4	0.5379	0.3624	−0.0838	0.026*	
O5	0.81234 (10)	0.13738 (12)	0.18938 (4)	0.0221 (2)	
C5	0.68105 (13)	0.41230 (16)	−0.01829 (5)	0.0198 (3)	
C6	0.72402 (13)	0.38397 (15)	0.03549 (5)	0.0178 (3)	
H6	0.8068	0.4308	0.0540	0.021*	
C7	0.64034 (12)	0.28344 (15)	0.06143 (5)	0.0160 (3)	
C8	0.65137 (13)	0.22643 (16)	0.11585 (5)	0.0158 (2)	
C9	0.76525 (12)	0.25249 (16)	0.16090 (5)	0.0167 (3)	
C10	0.81371 (13)	0.42143 (17)	0.17023 (5)	0.0177 (3)	
C14	0.51626 (12)	0.02212 (16)	0.16433 (5)	0.0161 (3)	
C15	0.50330 (16)	0.02643 (19)	0.25714 (5)	0.0262 (3)	
H15A	0.4507	−0.0773	0.2490	0.031*	
H15B	0.4537	0.0955	0.2800	0.031*	
C16	0.64444 (18)	−0.0127 (2)	0.28626 (6)	0.0386 (4)	
H16A	0.6977	0.0897	0.2930	0.058*	
H16C	0.6911	−0.0873	0.2646	0.058*	
H16B	0.6370	−0.0660	0.3203	0.058*	
C17	0.31674 (13)	0.06954 (16)	0.05885 (5)	0.0164 (3)	
C18	0.12943 (13)	0.00596 (17)	0.10920 (5)	0.0191 (3)	
C19	0.02392 (14)	0.12342 (19)	0.07961 (6)	0.0266 (3)	
H19A	0.0349	0.1279	0.0420	0.040*	
H19B	0.0376	0.2340	0.0953	0.040*	
H19C	−0.0690	0.0843	0.0824	0.040*	
C20	0.13131 (15)	0.01449 (19)	0.16902 (6)	0.0255 (3)	
H20A	0.1547	0.1270	0.1816	0.038*	
H20B	0.2002	−0.0629	0.1870	0.038*	
H20C	0.0402	−0.0151	0.1770	0.038*	
C21	0.11243 (16)	−0.16979 (18)	0.08904 (6)	0.0272 (3)	
H21A	0.1883	−0.2376	0.1072	0.041*	
H21C	0.1133	−0.1715	0.0506	0.041*	
H21B	0.0246	−0.2142	0.0963	0.041*	
O6A	0.9318 (4)	0.4474 (3)	0.20578 (19)	0.0224 (4)	0.809 (6)
C11A	0.7582 (4)	0.5688 (4)	0.1527 (3)	0.0194 (6)	0.809 (6)
H11A	0.6773	0.5851	0.1273	0.023*	0.809 (6)
C12A	0.8436 (3)	0.6940 (4)	0.17952 (12)	0.0211 (6)	0.809 (6)
H12A	0.8306	0.8101	0.1763	0.025*	0.809 (6)
C13A	0.9470 (3)	0.6138 (4)	0.21058 (12)	0.0242 (6)	0.809 (6)
H13A	1.0210	0.6668	0.2329	0.029*	0.809 (6)
O6B	0.7353 (13)	0.5546 (12)	0.1514 (8)	0.0194 (6)	0.191 (6)
C11B	0.929 (2)	0.4726 (19)	0.2027 (13)	0.0224 (4)	0.191 (6)
H11B	0.9978	0.4049	0.2228	0.027*	0.191 (6)
C12B	0.9271 (18)	0.6477 (18)	0.2006 (6)	0.0242 (6)	0.191 (6)
H12B	0.9966	0.7204	0.2175	0.029*	0.191 (6)
C13B	0.8072 (14)	0.691 (2)	0.1701 (6)	0.0211 (6)	0.191 (6)
H13B	0.7774	0.8017	0.1628	0.025*	0.191 (6)

### Atomic displacement parameters (Å^2^)

**Table:**

	*U*^11^	*U*^22^	*U*^33^	*U*^12^	*U*^13^	*U*^23^
Cl1	0.0375 (2)	0.01775 (17)	0.02629 (18)	0.00140 (14)	0.01670 (14)	0.00393 (13)
O1	0.0191 (5)	0.0232 (5)	0.0189 (5)	−0.0008 (4)	0.0000 (4)	−0.0042 (4)
N1	0.0155 (5)	0.0154 (5)	0.0146 (5)	0.0006 (4)	0.0022 (4)	−0.0001 (4)
C1	0.0157 (6)	0.0156 (6)	0.0155 (6)	0.0022 (5)	0.0014 (4)	−0.0012 (5)
O2	0.0148 (4)	0.0237 (5)	0.0185 (4)	−0.0018 (4)	0.0034 (3)	−0.0016 (4)
C2	0.0172 (6)	0.0141 (6)	0.0168 (6)	0.0033 (5)	0.0054 (5)	−0.0010 (5)
O3	0.0293 (5)	0.0167 (5)	0.0204 (5)	−0.0011 (4)	0.0027 (4)	0.0004 (4)
C3	0.0210 (6)	0.0181 (6)	0.0174 (6)	0.0047 (5)	0.0019 (5)	−0.0017 (5)
O4	0.0288 (5)	0.0186 (5)	0.0156 (4)	−0.0014 (4)	0.0072 (4)	−0.0002 (4)
C4	0.0305 (7)	0.0195 (7)	0.0150 (6)	0.0076 (6)	0.0061 (5)	0.0008 (5)
O5	0.0212 (5)	0.0219 (5)	0.0219 (5)	0.0017 (4)	−0.0003 (4)	0.0036 (4)
C5	0.0266 (7)	0.0141 (6)	0.0214 (6)	0.0045 (5)	0.0122 (5)	0.0022 (5)
C6	0.0186 (6)	0.0150 (6)	0.0207 (6)	0.0029 (5)	0.0061 (5)	0.0005 (5)
C7	0.0173 (6)	0.0145 (6)	0.0165 (6)	0.0041 (5)	0.0038 (5)	−0.0001 (5)
C8	0.0160 (6)	0.0149 (6)	0.0166 (6)	0.0016 (5)	0.0032 (5)	0.0000 (5)
C9	0.0140 (6)	0.0208 (7)	0.0156 (6)	0.0010 (5)	0.0039 (5)	0.0009 (5)
C10	0.0149 (6)	0.0230 (7)	0.0152 (6)	−0.0019 (5)	0.0021 (5)	0.0004 (5)
C14	0.0130 (6)	0.0186 (6)	0.0165 (6)	0.0001 (5)	0.0013 (4)	−0.0002 (5)
C15	0.0401 (8)	0.0247 (7)	0.0161 (6)	−0.0044 (6)	0.0112 (6)	0.0008 (5)
C16	0.0463 (10)	0.0455 (10)	0.0211 (7)	−0.0115 (8)	−0.0030 (7)	0.0079 (7)
C17	0.0149 (6)	0.0137 (6)	0.0203 (6)	0.0021 (5)	0.0025 (5)	−0.0009 (5)
C18	0.0133 (6)	0.0207 (7)	0.0239 (7)	−0.0016 (5)	0.0051 (5)	−0.0025 (5)
C19	0.0182 (7)	0.0284 (8)	0.0327 (8)	0.0033 (6)	0.0033 (6)	−0.0008 (6)
C20	0.0216 (7)	0.0316 (8)	0.0249 (7)	−0.0040 (6)	0.0091 (5)	−0.0031 (6)
C21	0.0283 (7)	0.0214 (7)	0.0340 (8)	−0.0049 (6)	0.0114 (6)	−0.0045 (6)
O6A	0.0205 (5)	0.0241 (9)	0.0202 (8)	−0.0030 (8)	−0.0043 (4)	0.0034 (10)
C11A	0.0118 (14)	0.0253 (9)	0.0207 (7)	−0.0007 (9)	0.0015 (12)	0.0004 (8)
C12A	0.0194 (16)	0.0205 (7)	0.0230 (14)	−0.0035 (13)	0.0019 (11)	0.0011 (9)
C13A	0.0259 (12)	0.0246 (15)	0.0199 (14)	−0.0089 (10)	−0.0031 (8)	0.0012 (9)
O6B	0.0118 (14)	0.0253 (9)	0.0207 (7)	−0.0007 (9)	0.0015 (12)	0.0004 (8)
C11B	0.0205 (5)	0.0241 (9)	0.0202 (8)	−0.0030 (8)	−0.0043 (4)	0.0034 (10)
C12B	0.0259 (12)	0.0246 (15)	0.0199 (14)	−0.0089 (10)	−0.0031 (8)	0.0012 (9)
C13B	0.0194 (16)	0.0205 (7)	0.0230 (14)	−0.0035 (13)	0.0019 (11)	0.0011 (9)

### Geometric parameters (Å, º)

**Table:**

Cl1—C5	1.7411 (13)	C15—H15A	0.9900
O1—C17	1.1973 (15)	C15—H15B	0.9900
N1—C1	1.3929 (15)	C16—H16A	0.9800
N1—C2	1.4059 (16)	C16—H16C	0.9800
N1—C17	1.4209 (16)	C16—H16B	0.9800
C1—C8	1.3651 (18)	C18—C21	1.5121 (19)
C1—C14	1.4943 (17)	C18—C19	1.5145 (19)
O2—C17	1.3253 (15)	C18—C20	1.5203 (19)
O2—C18	1.4919 (15)	C19—H19A	0.9800
C2—C3	1.3933 (17)	C19—H19B	0.9800
C2—C7	1.4053 (17)	C19—H19C	0.9800
O3—C14	1.2023 (16)	C20—H20A	0.9800
C3—C4	1.3859 (19)	C20—H20B	0.9800
C3—H3	0.9500	C20—H20C	0.9800
O4—C14	1.3329 (15)	C21—H21A	0.9800
O4—C15	1.4672 (16)	C21—H21C	0.9800
C4—C5	1.3959 (14)	C21—H21B	0.9800
C4—H4	0.9500	O6A—C13A	1.359 (3)
O5—C9	1.2240 (16)	C11A—C12A	1.418 (3)
C5—C6	1.3836 (13)	C11A—H11A	0.9500
C6—C7	1.3968 (13)	C12A—C13A	1.349 (3)
C6—H6	0.9500	C12A—H12A	0.9500
C7—C8	1.4463 (16)	C13A—H13A	0.9500
C8—C9	1.4816 (17)	O6B—C13B	1.357 (3)
C9—C10	1.4548 (19)	C11B—C12B	1.418 (4)
C10—C11A	1.357 (3)	C11B—H11B	0.9500
C10—C11B	1.357 (4)	C12B—C13B	1.348 (3)
C10—O6B	1.366 (3)	C12B—H12B	0.9500
C10—O6A	1.367 (2)	C13B—H13B	0.9500
C15—C16	1.497 (2)		
			
C1—N1—C2	108.06 (10)	H16A—C16—H16C	109.5
C1—N1—C17	125.70 (10)	C15—C16—H16B	109.5
C2—N1—C17	123.65 (10)	H16A—C16—H16B	109.5
C8—C1—N1	109.83 (11)	H16C—C16—H16B	109.5
C8—C1—C14	127.02 (11)	O1—C17—O2	129.43 (12)
N1—C1—C14	122.51 (11)	O1—C17—N1	122.44 (12)
C17—O2—C18	121.78 (10)	O2—C17—N1	108.11 (10)
C3—C2—C7	122.29 (12)	O2—C18—C21	109.35 (11)
C3—C2—N1	129.98 (12)	O2—C18—C19	109.57 (11)
C7—C2—N1	107.74 (10)	C21—C18—C19	113.13 (12)
C4—C3—C2	117.04 (12)	O2—C18—C20	101.33 (10)
C4—C3—H3	121.5	C21—C18—C20	111.39 (12)
C2—C3—H3	121.5	C19—C18—C20	111.40 (12)
C14—O4—C15	116.71 (10)	C18—C19—H19A	109.5
C3—C4—C5	120.71 (12)	C18—C19—H19B	109.5
C3—C4—H4	119.6	H19A—C19—H19B	109.5
C5—C4—H4	119.6	C18—C19—H19C	109.5
C6—C5—C4	122.72 (12)	H19A—C19—H19C	109.5
C6—C5—Cl1	118.40 (9)	H19B—C19—H19C	109.5
C4—C5—Cl1	118.87 (9)	C18—C20—H20A	109.5
C5—C6—C7	117.05 (11)	C18—C20—H20B	109.5
C5—C6—H6	121.5	H20A—C20—H20B	109.5
C7—C6—H6	121.5	C18—C20—H20C	109.5
C6—C7—C2	120.18 (11)	H20A—C20—H20C	109.5
C6—C7—C8	132.81 (11)	H20B—C20—H20C	109.5
C2—C7—C8	107.00 (10)	C18—C21—H21A	109.5
C1—C8—C7	107.37 (11)	C18—C21—H21C	109.5
C1—C8—C9	123.78 (11)	H21A—C21—H21C	109.5
C7—C8—C9	128.65 (11)	C18—C21—H21B	109.5
O5—C9—C10	122.45 (11)	H21A—C21—H21B	109.5
O5—C9—C8	121.01 (12)	H21C—C21—H21B	109.5
C10—C9—C8	116.51 (11)	C13A—O6A—C10	106.33 (19)
C11A—C10—C11B	100.6 (7)	C10—C11A—C12A	107.2 (3)
C11B—C10—O6B	110.0 (9)	C10—C11A—H11A	126.4
C11A—C10—O6A	109.6 (2)	C12A—C11A—H11A	126.4
O6B—C10—O6A	118.8 (7)	C13A—C12A—C11A	105.6 (3)
C11A—C10—C9	132.0 (2)	C13A—C12A—H12A	127.2
C11B—C10—C9	127.3 (7)	C11A—C12A—H12A	127.2
O6B—C10—C9	122.2 (6)	C12A—C13A—O6A	111.3 (3)
O6A—C10—C9	118.21 (14)	C12A—C13A—H13A	124.4
O3—C14—O4	126.20 (12)	O6A—C13A—H13A	124.4
O3—C14—C1	123.44 (12)	C13B—O6B—C10	106.7 (13)
O4—C14—C1	110.32 (11)	C10—C11B—C12B	106.0 (11)
O4—C15—C16	110.56 (12)	C10—C11B—H11B	127.0
O4—C15—H15A	109.5	C12B—C11B—H11B	127.0
C16—C15—H15A	109.5	C13B—C12B—C11B	106.8 (15)
O4—C15—H15B	109.5	C13B—C12B—H12B	126.6
C16—C15—H15B	109.5	C11B—C12B—H12B	126.6
H15A—C15—H15B	108.1	C12B—C13B—O6B	110.3 (17)
C15—C16—H16A	109.5	C12B—C13B—H13B	124.9
C15—C16—H16C	109.5	O6B—C13B—H13B	124.9
			
C2—N1—C1—C8	0.90 (14)	C8—C9—C10—O6A	−170.0 (3)
C17—N1—C1—C8	−161.26 (11)	C15—O4—C14—O3	3.66 (19)
C2—N1—C1—C14	−170.51 (11)	C15—O4—C14—C1	−173.96 (11)
C17—N1—C1—C14	27.33 (19)	C8—C1—C14—O3	−116.54 (15)
C1—N1—C2—C3	178.85 (13)	N1—C1—C14—O3	53.33 (18)
C17—N1—C2—C3	−18.5 (2)	C8—C1—C14—O4	61.16 (17)
C1—N1—C2—C7	−1.16 (13)	N1—C1—C14—O4	−128.97 (12)
C17—N1—C2—C7	161.45 (11)	C14—O4—C15—C16	88.55 (15)
C7—C2—C3—C4	0.00 (19)	C18—O2—C17—O1	6.0 (2)
N1—C2—C3—C4	179.99 (12)	C18—O2—C17—N1	−175.66 (10)
C2—C3—C4—C5	−0.72 (19)	C1—N1—C17—O1	−169.19 (12)
C3—C4—C5—C6	1.7 (2)	C2—N1—C17—O1	31.29 (19)
C3—C4—C5—Cl1	−177.20 (10)	C1—N1—C17—O2	12.35 (17)
C4—C5—C6—C7	−1.74 (19)	C2—N1—C17—O2	−147.17 (11)
Cl1—C5—C6—C7	177.11 (9)	C17—O2—C18—C21	58.82 (15)
C5—C6—C7—C2	0.99 (18)	C17—O2—C18—C19	−65.71 (15)
C5—C6—C7—C8	179.53 (13)	C17—O2—C18—C20	176.51 (11)
C3—C2—C7—C6	−0.15 (19)	C11A—C10—O6A—C13A	0.9 (6)
N1—C2—C7—C6	179.86 (11)	C11B—C10—O6A—C13A	14 (13)
C3—C2—C7—C8	−179.03 (12)	O6B—C10—O6A—C13A	−4.8 (11)
N1—C2—C7—C8	0.98 (13)	C9—C10—O6A—C13A	−174.7 (3)
N1—C1—C8—C7	−0.28 (14)	C11B—C10—C11A—C12A	−3.5 (19)
C14—C1—C8—C7	170.64 (12)	O6B—C10—C11A—C12A	150 (7)
N1—C1—C8—C9	−175.47 (11)	O6A—C10—C11A—C12A	−1.5 (6)
C14—C1—C8—C9	−4.5 (2)	C9—C10—C11A—C12A	173.4 (2)
C6—C7—C8—C1	−179.12 (13)	C10—C11A—C12A—C13A	1.4 (5)
C2—C7—C8—C1	−0.44 (14)	C11A—C12A—C13A—O6A	−0.8 (5)
C6—C7—C8—C9	−4.2 (2)	C10—O6A—C13A—C12A	0.0 (5)
C2—C7—C8—C9	174.43 (12)	C11A—C10—O6B—C13B	−24 (5)
C1—C8—C9—O5	41.90 (19)	C11B—C10—O6B—C13B	3 (3)
C7—C8—C9—O5	−132.21 (14)	O6A—C10—O6B—C13B	6.5 (19)
C1—C8—C9—C10	−136.08 (13)	C9—C10—O6B—C13B	176.0 (10)
C7—C8—C9—C10	49.81 (18)	C11A—C10—C11B—C12B	0 (3)
O5—C9—C10—C11A	−162.5 (4)	O6B—C10—C11B—C12B	−5 (3)
C8—C9—C10—C11A	15.5 (4)	O6A—C10—C11B—C12B	−167 (15)
O5—C9—C10—C11B	14 (2)	C9—C10—C11B—C12B	−176.6 (12)
C8—C9—C10—C11B	−168 (2)	C10—C11B—C12B—C13B	4 (3)
O5—C9—C10—O6B	−157.6 (11)	C11B—C12B—C13B—O6B	−2 (3)
C8—C9—C10—O6B	20.4 (11)	C10—O6B—C13B—C12B	−1 (2)
O5—C9—C10—O6A	12.0 (4)		

### Hydrogen-bond geometry (Å, º)

**Table:**

*D*—H···*A*	*D*—H	H···*A*	*D*···*A*	*D*—H···*A*
C3—H3···O1	0.95	2.45	2.9774 (16)	115
C13*A*—H13*A*···O5^i^	0.95	2.37	3.187 (3)	144
C19—H19*A*···O1	0.98	2.49	3.0985 (17)	120
C21—H21*C*···O1	0.98	2.49	3.0615 (17)	117

Symmetry code: (i) −*x*+2, *y*+1/2, −*z*+1/2.
